# Herbal Tea for the Management of Pharyngitis: Inhibition of *Streptococcus pyogenes* Growth and Biofilm Formation by Herbal Infusions

**DOI:** 10.3390/biomedicines7030063

**Published:** 2019-08-24

**Authors:** Niluni M. Wijesundara, H. P. Vasantha Rupasinghe

**Affiliations:** 1 Department of Biology, Faculty of Science, Dalhousie University, Halifax, NS 3H 4R2, Canada; 2 Department of Animal Science, Faculty of Animal Science and Export Agriculture, Uva Wellassa University, Badulla 90 000, Sri Lanka; 3 Department of Plant, Food, and Environmental Sciences, Faculty of Agriculture, Dalhousie University, Truro, NS B2N 5E3, Canada; 4 Department of Pathology, Faculty of Medicine, Dalhousie University, Halifax, NS B3H 4R2, Canada

**Keywords:** herbal plants, hot water infusions, phytochemicals, natural health product, *Streptococcus pyogenes*, mass spectrometry

## Abstract

Herbal teas are becoming popular as functional beverages due to their various health promotional properties. This study aimed at assessing 13 hot water infusions (HWIs) from different herbs against streptococcal pharyngitis (strep throat). Licorice root exhibited the lowest minimum inhibitory concentrations (MIC) of 1.56 mg/mL, followed by barberry root, thyme, and oregano flowering shoots, with a MIC of 3.13 mg/mL. At their respective minimum bactericidal concentrations (MBC), licorice showed the bactericidal effect on *S. pyogenes* within 12 h after exposure while others need 24 h for a similar outcome. The HWIs exhibited inhibitory activity on biofilm formation, ranging from 1.56 to 6.25 mg/mL, which confirmed by ruptured cells or clusters of dead cell debris observed in scanning electron microscope (SEM). Overall, non-toxic concentrations of efficacious HWIs from licorice root, barberry root, thyme, and oregano flowering shoots may provide potential sources for developing herbal teas or biomedicine for the management of *S. pyogenes* infections.

## 1. Introduction

For centuries, the therapeutic properties of various spices and herbal plants have been used to treat several bacterial infections. Streptococcal pharyngitis (strep throat), an acute infection of the nasopharynx and oropharynx, is one of the common upper respiratory infections, primarily caused by *Streptococcus pyogenes.* It accounts for more than 37% of all diagnosed sore throat cases in children and up to 5–10% in adults reports millions of cases per year worldwide [1,2].

Investigation of natural antimicrobial agents as alternatives to synthetic counterparts have received significant attention from researchers and natural health product industry. Therefore, plant-derived new natural antimicrobial agents have been explored against various infectious bacteria, including *S. pyogenes.* It has been presumed that herbal remedies and formulations are moderately efficacious but safer from side effects in contrast to most pharmaceutical agents [3]. A World Health Organization survey reported that around 70–80% of the world population use herbal remedies as their primary health care [4]. Usage of complementary and alternative therapies in Canada has increased at an exponentially growing pace in recent years, and the estimated use of alternative or herbal remedies in place of conventional medicine is nearly 30% of the total population in the United States [5].

The use of herbal plants for various medicinal purposes by traditional healers in North America has been reported. Interestingly, over 2500 species of plants have been used by both Native Americans and Americans of European origin in their traditional medicine systems [6]. The First Nations have used the fresh plant parts or their extracts of herbal plants such as slippery elm, sage, barberry, oregano, thyme, licorice, and echinacea, and Canadian folk remedial applies for sore throat conditions. Based on the plant used, type of infection, and the situations of the patient is being treated the preparation of herbal remedies are different such as infusions (hot teas), decoctions (boiled teas), macerations (cold-soaking), or tinctures (solvent/water extracts and distillations) [7].

The rapidly expanding natural health product industries keep introducing new herbal supplements, functional food, and herbs-based energy drinks led by the increased demand for the use of medicinal remedies from consumers. Drinking herbal tea is gaining popularity as one of the most pleasurable beverages as well as an efficient herbal remedy due to its health promotional properties. Herbal teas have been identified and approved as a natural and nonprescription drug in some countries, including Canada, as a remedy for oral and pharyngeal mucosal irritations with a dry cough.

Due to the potential side effects of long-term administration of traditional herbal medicines for the management of streptococcal pharyngitis, it is necessary to have proper clinical laboratory investigations on their activity and safety compared with synthetic antibiotics. However, hot water infusions (HWIs) of selected plant parts of the current study (Table 1) have not been reported or limited with emphasizing both growth and biofilm inhibition of *S. pyogenes.* Therefore, this study aimed to identify the potential herbals with antibacterial activity and antibiofilm efficacy of 13 HWIs from different parts of herbal plants against three *S. pyogenes* strains, to develop specialty herbal tea for relieving pain and other complications due to streptococcal pharyngitis.

## 2. Materials and Methods

### 2.1. Chemicals and Reagents

3-(4, 5-Dimethylthiazol-2-yl)-2, 5-diphenyltetrazolium bromide (MTT) was purchased from Life Technologies (Burlington, ON, Canada). Bacteriological agar and brain heart infusion (BHI) media were purchased from Oxoid Ltd. (Nepean, ON, Canada). Dimethyl sulfoxide (DMSO), penicillin G sodium salt, phosphate-buffered saline (PBS), sodium chloride (≥99.0%, ACS reagent), and other chemicals were obtained from Sigma-Aldrich (Oakville, ON, Canada).

### 2.2. Collection of Plant Materials

Eleven different medicinal plants which were commonly used in the Canadian indigenous medicine were considered in the present study. Their common names, scientific names, and specific parts used are summerized in Table 1 were selected. Geranium (Voucher No: 13010), Sage (Voucher No: 13011), oregano (Voucher No: 13012), and thyme (Voucher No: 13013) were collected from the university’s herbal garden, Faculty of Agriculture, Dalhousie University, at GPS location of 45°22’23.3”N and 63°15’45.2”W during flowering period. Purple coneflower/Echinacea (Voucher No: 13009), was collected at GPS location of 45°22’20.8”N and 63°15’43.8”W. A taxonomist was authenticated the herbal plants and specimens were deposited in the A.E. Roland herbarium, Department of Plant, Food, Environmental Sciences, Faculty of Agriculture, Dalhousie University, Canada. Canadian ginger and clove flower buds were purchased from the local and supermarket. Fresh plant parts were washed, cut into small pieces, and dried at 50 °C. Ground powders were stored at −80 °C in airtight containers. Barberry root, slippery elm inner bark, licorice root, and olive leaves were obtained as a powder from Mother Earth Natural Health Inc. (Ottawa, ON, Canada).

### 2.3. Preparation of Hot Water Infusions (HWIs)

Dried powder of herbal plant parts was infused with distilled water in 1:10 (*w*/*v*) ration in a boiling water bath (ISOTEMP-205, Fisher Scientific, Ottawa, ON, Canada) for 10 min. After infusion period, mixtures were filtered. Filtrates were frozen overnight and freeze dried at −20 °C using a freeze dryer (Kinetics, FTS Systems Inc., Stone Ridge, NY, USA) for 24–48 h. The dried samples were scraped out and were stored in labeled sterile screw-capped amber bottles, in the freezer at -80 °C until used for further analysis.

### 2.4. Characterization of Phytochemicals in HWIs

#### 2.4.1. Determination of Total Phenolic and Total Carotenoids Contents

The total phenolic contents and total carotenoids content were assessed for all the HWIs, and results were previously published by authors [8].

#### 2.4.2. Characterization of Phytochemical Profile

Potential phytochemicals present in HWIs were characterized using ultra-performance liquid chromatographic-electrospray ionization-tandem mass spectrometry (UPLC-ESI-MS/MS) as described in our previous study [9]. Briefly, UPLC was directly interfaced with a High Definition MS System (Waters Xevo TQ-Smicro, Waters Corporation, Milford, USA) with an electrospray ionization (ESI) source operating in negative ion mode with ionization conditions of capillary voltage of 2.0 V, sampling cone voltage of 25.0 V and extraction cone voltage of 3.5 V. The optimal temperatures (150 °C of source temperature and 450 °C of desolvation gas temperature) and optimal gas flows (100 L/h of cone gas flow and 1000 L/h of desolvation gas flow) were maintained. Full-scan mass acquisitions in negative ion mode were made by scanning the *m/z* range of 100–1100 Da. Data were collected in centroid mode. Masses were corrected using an external reference (Lock-Spray™) comprising a C_18_ column (UPLC^®^ BEH C_18_, Waters Corporation, USA). The filtered samples were auto injected into the column. A mixture of 0.1% formic acid in water (solvent A) and 0.1% formic acid in acetonitrile (Solvent B) was used as the mobile phase. The total run time was 12 min (2 min (83.5% A), 2.6 min (83.0% A), 3.1 min (82.5% A), 4 min (81.5% A), 4.7 min (80.0% A), 6.6 min (20.0% A), 8.2 min (20.0% A), and 12 min (94.0%)), with 94.0% of solvent A flow of 0.3 mL/min. The deprotonated (M–H^+^)^−^ ions identified using the full scan mode was used to generate selected ion monitoring (SIM) channels (51 channels). The samples were run in SIM mode to confirm the abundance of each recognized compounds matching to their retention times of SIM mode and full scan mode. These identified phytochemicals were confirmed with the existing literature (Table 1).

### 2.5. Bacteria and Growth Conditions

Two American Type Culture Collection (ATCC) of *S. pyogenes* (ATCC 19615 and ATCC 49399), as well as a pharyngeal isolate from a streptococcal pharyngitis patient (Queen Elizabeth II Health Sciences Centre, Halifax, NS, Canada) were used.

Following the manufacturer’s instructions, inoculums were prepared and stored at −80 °C in 1:1 (*v*/*v*) of brain heart infusion (BHI) broth: 40% glycerol. Strains were cultured on BHI agar plates, were maintained for seven days at 37 °C. For experiments, a few colonies were inoculated in BHI broth, were incubated at 37 °C for about 16 h and standardized with saline water (0.85% NaCl, pH = 7.0 ± 0.1) according to the previously reported method [10].

### 2.6. Anti-Bacterial Activity

#### 2.6.1. Screening for Inhibitory Antibacterial Effects of HWIs

The spot-on-the-lawn method was used for initial screening to verify the antibacterial effects of HWIs, with ATCC 19615 and ATCC 49399 bacterial cultures. The samples were spotted, plates were incubated at 37 °C for 24–48 h and examined for the zone of inhibitions.

#### 2.6.2. Determination of Minimum Inhibitory Concentrations (MICs)

Effect of HWIs on bacterial growth was assessed using the standard micro broth-dilution method recommended by the Clinical and Laboratory Standards Institute [11]. Briefly, 100 µL volumes of diluted bacterial suspensions (10^6^ CFU/mL) in BHI broth were incubated with the same amount of serial two-fold diluted HWIs (0.2 to 50 mg/mL), penicillin G (4.0 × 10^−4^ to 0.5 μg/mL), and BHI media (diluent control). The assay was carried out in 96-well microplate, and bacteria growth was measured as absorbance at OD 600 nm after 24 h incubation (37 °C). The MIC values corresponded to the lowest concentration of test compounds that inhibiting visible bacterial growth or showing a significant change of absorbance compared to the growth of control (in the spectrophotometric method) were recorded.

#### 2.6.3. Determination of Minimum Bactericidal Concentrations (MBCs)

MBCs were determined by subculturing 15 μL from non-turbid wells on BHI agar plate, and colony growth was observed after 24 h incubation at (37 °C). The MBC value is the lowest concentration where no visible colony growth was observed, compared to the control.

#### 2.6.4. Time-Kill Curves

The time taken to show a bactericidal activity by HWIs was measured using time-kill curves as a previously described method [10]. Briefly, the bacterial count was enumerated in 3 h intervals over 24 h incubation periods at 37 °C with different concentrations of licorice root, oregano flowering shoot, thyme flowering shoot HWIs (their own 1/2 × MIC, MIC, and 2 × MIC). BHI media alone with bacteria was used as a diluent control, and penicillin G was as the positive control. The assays were performed in triplicate, and the results were expressed as *log* CFU/mL.

### 2.7. Anti-Biofilm Formation Activity

#### 2.7.1. Determination of Minimum Biofilm Inhibitory Concentrations (MBIC) and Biofilm Quantification by MTT Assay

The effect of HWIs on biofilm formation of *S.* pyogenes was examined using a 3-[4–dimethyl-2-thiazolyl]-2, 5-diphenyl-2H-tetrazolium-bromide (MTT) assay as described previously [10]. As in the MIC assay, two-fold serial dilutions of four HWIs and penicillin G were prepared in 96-well plates and were inoculated with 100 μL of 1 × 10^6^ CFU/mL bacterial suspensions. After three days of incubation at 37 °C, the plates were emptied by flipping them over to remove the planktonic bacteria. Fresh BHI broth (100 μL) supplemented with 10 μL of 12 mM MTT was then added into each well, followed by incubation for 3 h at 37 °C. DMSO (50 μL) was added after the careful removal of 85 μL of BHI broth from each well. Biofilm formation was calorimetrically quantified by measuring reduction ability of tetrazolium salt (yellow) into a formazan (purple) by the activity of dehydrogenase enzymes in surviving bacteria in biofilms. Absorbance at 540 nm was measured using a microplate reader (EpochTM, Biotek, Winooski, VT, USA). Percentage inhibitions were calculated as follows: [1 − (A_540_ Treatment /A_540_ Control)] × 100. The minimum biofilm inhibition concentration (MBIC) was defined as the lowest concentration.

#### 2.7.2. SEM Visualization of Biofilms

To examine the effects of selected HWIs on morphology, treated bacterial cells with HWIs at their respective sub-MIBCs were fixed as described in Wijesundara and Rupasinghe [10]. Briefly, treated cells were centrifuged, washed with PBS, and fixed in 0.1 M sodium cacodylate buffer (pH 7.2) containing 2% glutaraldehyde (2 h) and then in 4% osmium tetroxide (4 h). Biofilms were then dehydrated using gradient series of ethanol (35%; 50%; 75%; 90%; 100%) and hexamethyldisilazane/ethanol gradient series (25:75; 50:50; 75:25; 100:0%). The samples were air-dried for 2 h under the fume hood and were mounted on aluminum sputters. Then, sputters were coated with gold-palladium (15 nm) and were visualized under SEM (Hitachi FEG-SEM 4700, Hitachi Ltd., Tokyo, Japan) using operational conditions of 10 kilovolts (kV) of acceleration voltage, 14–16 microamps (µA) of emission current, 10–12 mm working distance and the analysis lens mode. Micrographs were captured at different magnifications. The experiment was performed in triplicates for three independent times.

### 2.8. Statistical Analysis

The complete randomized design was used, and all the experiments were performed in triplicates and three independent times. One-way analysis of variance (ANOVA) was performed using Minitab statistical software (Version 17.0, Minitab Inc., State College, PA, USA). Tukey’s test was used to determine the differences among treatments, and significant differences were defined as *p* < 0.05, and the results were expressed as the mean ± standard deviation. The time to kill curves were plotted using GraphPad Prism version 5.0 for Windows (GraphPad Software, La Jolla, CA, USA).

## 3. Results

### 3.1. Characterization of HWIs Using UPLC-ESI-MS/MS

**Figure 1:**
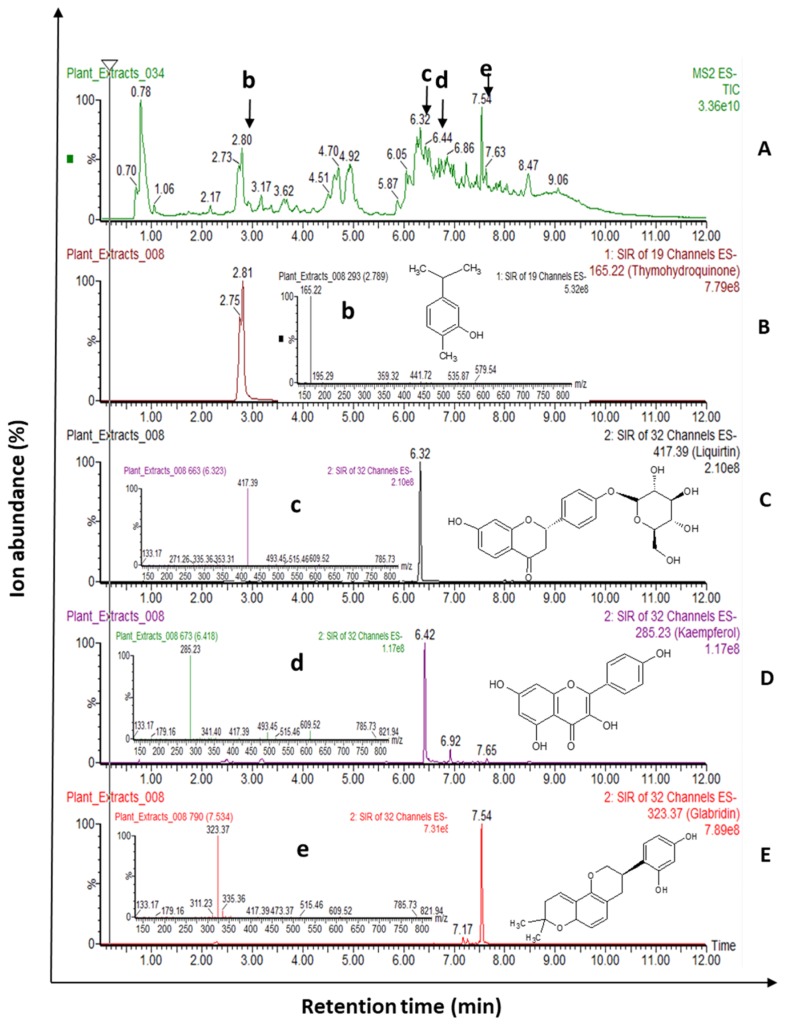
The full scan mode total ion chromatogram of licorice root hot water infusion from a negative mode of UPLC-ESI-MS/MS analysis (**A**). The SIM channels (**B**–**E**) and mass spectra of the full-scan of selected four major phytochemicals; thymohydroquinone (b), liquiritin (c), kaemferol (d), and glabridin (e) are shown for *m/z* of deprotonated ions of 165.22, 417.39, 285.23, and 323.97, respectively. The identified phytochemical name and chemical structure are presented. SIM: Selective ion monitoring scan; TIC: Total ion chromatograms, UPLC-ESI-MS/MS: Ultra-performance liquid chromatographic-electrospray ionization-tandem mass spectrometry.

We have previously reported the total phenolic content, and total carotenoid content of these HWIs [8]. Potential phytochemicals found in HWIs expressed with their deprotonated molecular mass and retention time ((M–H^+^)^−^, RT) were summarized in Table 1. The full-scan mode total ion chromatograms, SIM scan channels, mass spectrum, and molecular structure of the selected major phytochemicals identified by UPLC-ESI-MS/MS of most effective HWI, licorice root, are presented in Figure 1. Furthermore, UPLC-ESI-MS/MS identification results of barberry root, oregano flowering shoots, and thyme flowering shoots are included in supplementary figures section (Figures S1–S3).

### 3.2. HWIs Inhibits S.Pyogenes Planktonic Growth

**Figure 2:**
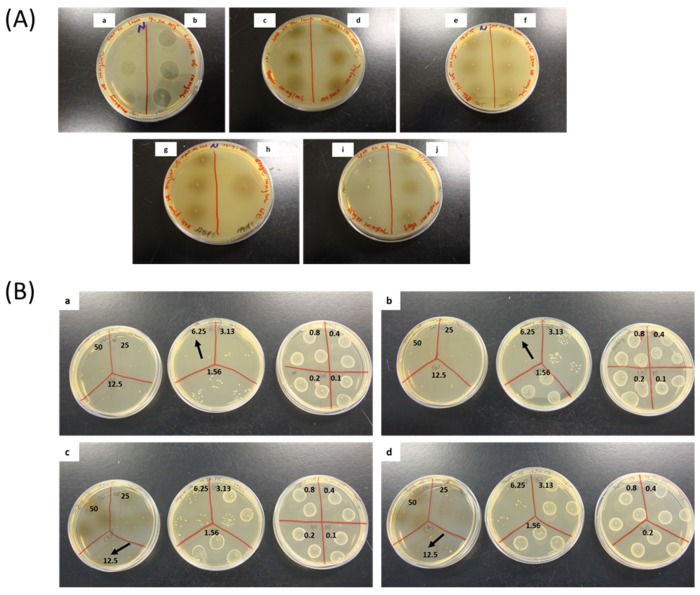
(**A**) Antibacterial effect of HWIs (100 mg/mL) spotted on brain heart infusion (BHI) agar plates inoculated with the *Streptococcus pyogenes* ATCC 19615. HWIs: Hot water infusions; a: barberry roots; b: licorice roots; c: thyme flowering shoots; d: oregano flowering shoots; e: echinacea leaves; f: echinacea stems; g: echinacea flowers; h: clove flower buds; i: ginger rhizomes, and j: sage leaves. (**B**) Sub-culturing for minimum bactericidal concentration on BHI agar plates of four most efficient hot water infusions against *S. pyogenes* ATCC 19615 followed by the micro-broth dilution assay. a: licorice roots; b: barberry roots; c: oregano flowering shoots; d: thyme flowering shoots.

The antibacterial activities of 13 HWIs have assessed against three strains of *S. pyogenes* (ATCC 19615, ATCC 49399 and a clinical isolate) and MIC and MBC values shown in Table 2. Zone of inhibitions around the spotted HWIs were detected in the initial screening and shown in Figure 2A. The HWI of licorice root exhibited greater activity against *S. pyogenes* planktonic growth with the lowest MIC of 1.56 mg/mL followed by barberry root, thyme, and oregano flowering shoots, as indicated by the relatively lower MIC of 3.13 mg/mL. Colony growth of sub-cultured four HWIs on BHI agar, which demonstrated significant distinct bactericidal effects shown in Figure 2B.

### 3.3. Time to Kill Analysis of HWIs Against S. Pyogenes

**Figure 3:**
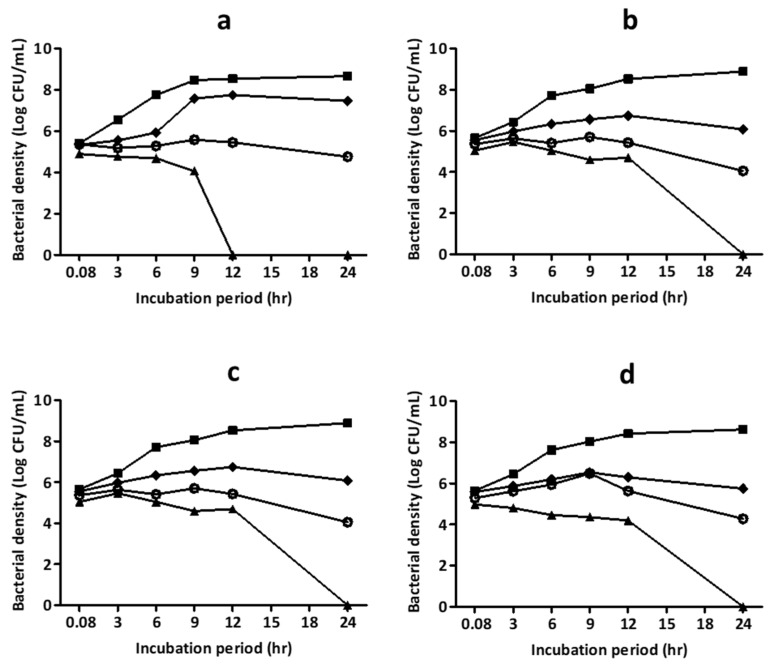
Time-kill curves of the hot water infusions of (**a**) licorice root, (**b**) barberry root, (**c**) oregano flowering shoots, and (**d**) thyme flowering shoots on the growth of *Streptococcus pyogenes* ATCC 19615. A viable count was performed for different folds of their respective minimum inhibitory concentrations (MICs) at 0.08, 3, 6, 9, 12, and 24 h incubation time points. ▲ = 2 × MIC; ◯ = MIC; ◆ = ½ × MIC; ■ = BHI media (diluent) control. Each data point represents mean from three independent experiments performed in triplicate.

HWIs from licorice roots, barberry roots, thyme flowering shoots, and oregano flowering shoots were selected for time-kill analysis based on their significantly low MIC and MBC values. Time taken to achieve 99.99% planktonic *S. pyogenes* kill by the HWIs were assessed, and results are shown in Figure 3. Results showed that licorice HWI exhibits the complete bactericidal effect on *S. pyogenes* within 12 h after exposure (Figure 3), whereas barberry, thyme, and oregano required a longer time of 24 h, at their respective MBC. No regrowth was observed after an additional 24 h incubation.

### 3.4. HWIs Possesses Anti-Biofilm Formation Activity

The HWIs were shown biofilm inhibition activities at the range of 1.56 to 6.25 mg/mL concentrations where licorice root infusion had the most active antibiofilm activity among the tested HWIs. The inhibition effects of sub-inhibitory concentrations of HWIs of licorice root, barberry root, oregano flowering shoots, and thyme flowering shoots on biofilm formation over 72 h incubation (Minimum biofilm inhibitory concentration; MBIC) of the three *S. pyogenes* strains were quantified by MTT staining (Table 3).

### 3.5. HWIs Cause Morphological Changes of S. pyogenes Biofilms

**Figure 4:**
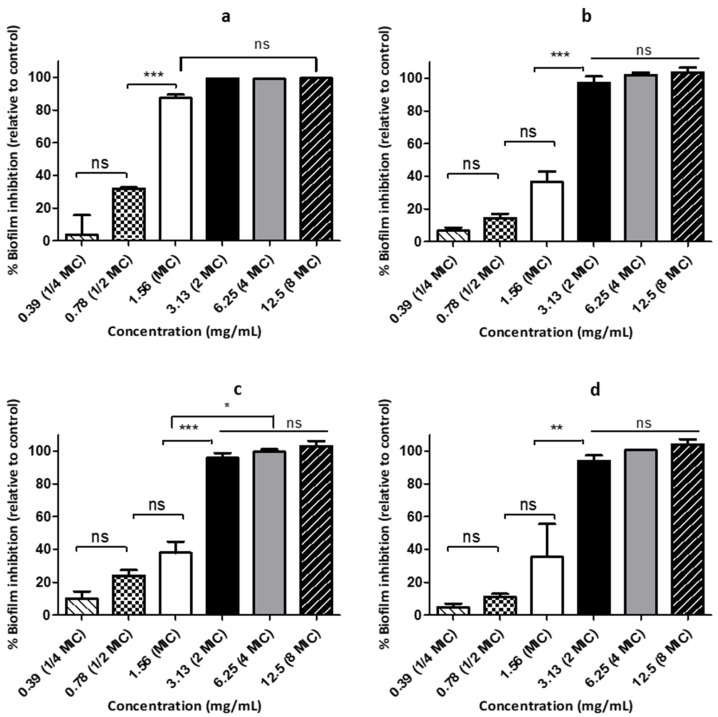
Inhibition of biofilm formation of *Streptococcus pyogenes* 19615 by hot water infusions at various concentrations. (**a**) licorice roots, (**b**) barberry roots, (**c**) oregano flowering shoots, and (**d**) thyme flowering shoots. Each data point represents mean ± SD from three independent experiments performed in triplicate.

**Figure 5:**
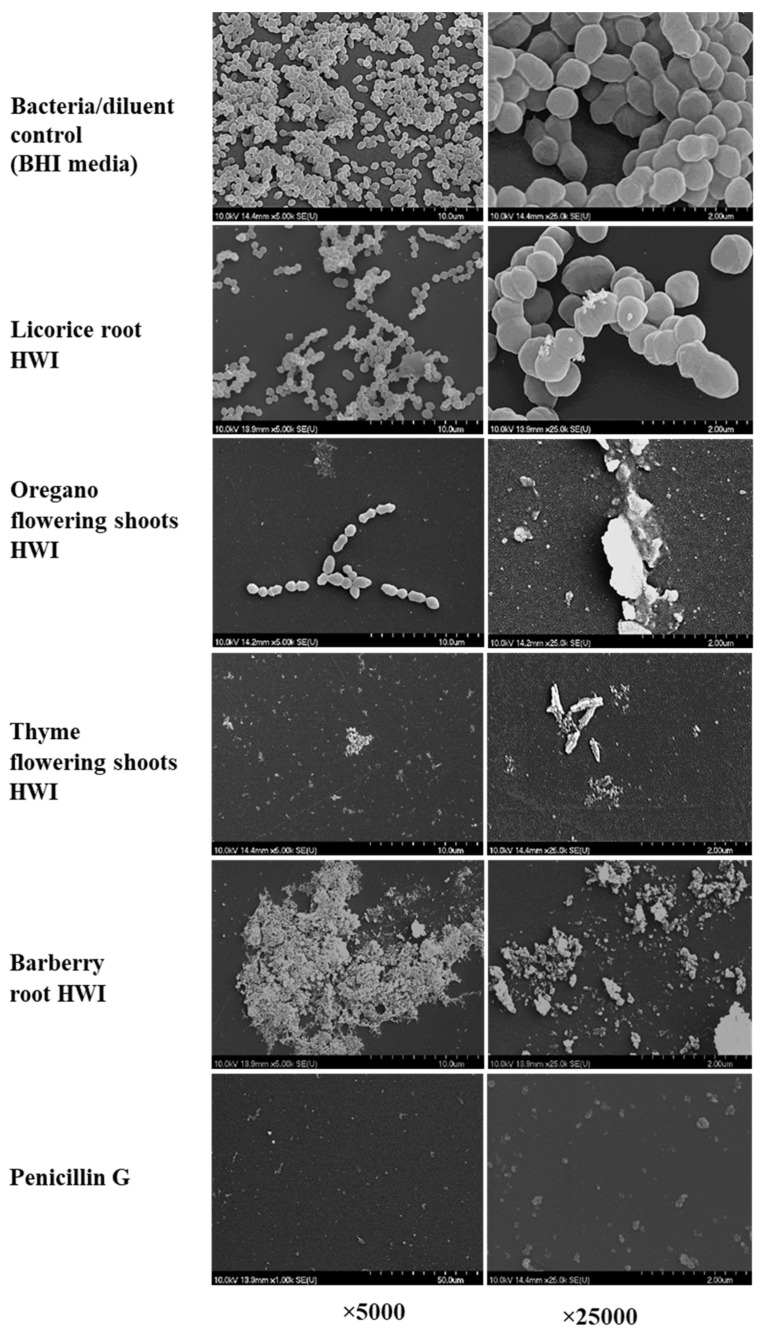
Scanning electron micrographs of biofilms of *Streptococcus pyogenes* formed on the microplate surface with the presence of hot water infusions of licorice roots, oregano flowering shoots, thyme flowering shoots, barberry root and penicillin G at their sub-inhibitory concentrations. HWI, hot water infusion.

The inhibition of biofilm was significantly effective, along with four HWIs when compared to controls, as shown in Figure 4. Mainly, biofilm reduction by 1/2 × MIC and MIC values of HWIs was about 87.4% and 99.1% for licorice root, 36.6% and 97.0% for barberry root, 37.9% and 95.8% for oregano, and 35.3% and 94.0% for thyme flowering shoot, respectively. Surface structure and morphology changes of biofilms formed with or without HWIs treatment at their sub-inhibitory MIBC, *S. pyogenes* ATCC 19615 are shown in Figure 5. The SEM analysis revealed that the HWIs cause noticeable cellular lysis and morphological alterations compared to untreated cells. Biofilms of untreated control showed a typical multi-layer bacterial colony growth while a significant reduction in microcolonies was observed in HWI-treated samples. Even at sub-inhibitory concentrations, penicillin G eradicated biofilm leaving a few of dead cell debris. Interestingly, HWIs of thyme and oregano were also showed significant destruction of biofilm by leaving either a few bacteria or cell debris. Dead cell debris resulted from substantial biofilm inhibition by barberry root HWI treatment resulting in a cluster, as shown in Figure 5. However, active disruption morphologies of cells such as ruptured shape were observed. Therefore, our findings provide evidence that HWIs has an intense antimicrobial action against *S. pyogenes* biofilm formation.

## 4. Discussion

Herbaceous plants are a source of complementary and alternative remedies to conventional medications in treating several bacterial infectious diseases, including streptococcal pharyngitis (strep throat). Previous studies have demonstrated that numerous herbal extracts could act as antibacterial agents against *S. pyogenes* [10,34,35,36,37,38]. In the present study, we focused on 13 HWIs, known to Canadian traditional and indigenous healers who used them against sore throat. The goal is to identify herbal plants as sources for specialty herbal tea to use in the management of streptococcal pharyngitis. Furthermore, we identified the major bioactive components in those HWIs that may contribute to their overall antimicrobial activities against *S. pyogenes.*

Spices and herbs (flavor foods) comprise a large number of phytochemicals [39], which have been revealed to possess antimicrobial properties. For example, anti-*S. pyogenes* ability of phenolic compounds [40,41], flavonoids [35], alkaloids [42], terpenoids [43], and tannins [35] have been reported. Qualitative UPLC analysis in the present study for HWI revealed the presence of polyphenols, flavonoids, alkaloids, terpenoids, steroids, and tannins. The phytochemical constituents of the different plants, as well as different parts of the same plant, showed phytochemical diversity, which partially explains the different antimicrobial potential established as a range of MIC and MBC values.

When comparing MIC and MBC values of the present study, licorice root, barberry root, oregano flowering shoot, and thyme flowering shoot infusions were identified as the most efficient HWIs. Similar antimicrobial potential of various varieties of thyme and oregano from different countries was also reported against *Streptococcus* species [43,44]. The Anti-bacterial efficacy of licorice root infusion was the most active extracts which showed the lowest MIC. Although HWIs of barberry, thyme, and oregano showed a concentration- and time-dependent bacteriostatic effect, a significant bactericidal effect was observed in licorice root infusion within 12 h of exposure at the concentration of 2 × MIC. The existence of antibacterial activities could be due to one or a few of phytochemicals specific to particular plant species or a particular part of a plant used. The chemical characterization of licorice infusion suggested the presence of specific phytochemicals such as glycyrrhizin, glabridin, naringenin, asparagine, and 5-methoxyhydnocarpin. Previous studies have also demonstrated that isoflavonoid compounds, such as glabridin, glycyrrhizin, glabriol, and hispaglabridin in the extracts purified from licorice roots, can act as bactericidal agents against various microorganisms [45].

Biofilm formation in *S. pyogenes* infections is one of the significant defensive mechanisms during pharyngitis infections where microbial cells tightly arranged and covered with extra polymeric substances [46,47]. Usually, biofilms make bacteria more resistant to antibiotics than their planktonic cells. Several other studies have shown that extractions or infusions from medicinal plants inhibit the biofilm formation in different bacteria, including *S. pyogenes* [41,48,49]. Our findings suggest that these HWIs of licorice roots, barberry roots, oregano, and thyme flowering shoots could be used against drug resistant-*S. pyogenes*. The present study investigated morphological changes such as structural alterations of *S. pyogenes* after exposure to the effective infusion treatments at their sub-MIBCs. The destruction of bacterial biofilms and cells shown in the SEM images are compatible with biofilm quantification findings of the MTT assay. Similar SEM observations against *S. pyogenes* have been reported [40,50].

Due to the recognition of significant healing power in traditional medicine systems, herbal medicines from indigenous pharmacopeia are being used as home remedies. On the other hand, public perception of using functional foods as biomedicines has begun to expand, especially in the Western world. Therefore, opportunities exist for innovation of efficacious, safe, and convenient herbal products as functional foods/biomedicines such as herbal teas and herbal tonics. However, some of the pharmacologically active compounds in herbs may cause side effects; thus, proper ethnomedical verification of efficacy and safety is required. On the other hand, no universal regulatory system ensures the efficacy and safety of plant remedies. Moreover, different countries have their policies and classification for natural health products. For example, herbal teas are categorized under “herb and plant-based remedies” in the Licensed Natural Health Products Database (LNHPD) in Canada and trade as an over-the-counter (OTC) product according to the report by consumer health product Canada [51], while herbal teas are traded as a dietary supplement in the United States. Therefore, extensive evidence for the in vitro, in vivo, or clinical efficacies, as well as pharmacological mechanisms of their phytochemical constituents, is necessary for the new product development.

Destruction of the cell wall, loss of cell membrane integrity due to disruption of phospholipid bilayer and inhibition of cell wall, membrane, protein, and DNA/RNA synthesis are considered as potential mechanisms of phytochemicals on their antibacterial and antibiofilm activities. However, since these HWIs contain a large number of different phytochemicals they may responsible for these activities through different mechanisms insole or combine. However, the present study does not have sufficient evidence to conclude the exact mechanism/s of these herbal extracts. Among the four most efficacious extracts identified, only licorice HWI shows moderately quick (12 h) bactericidal effect at its MBC value. The SEM images show clear cell destructions by the four HWIs when the bacterial cultures were exposed to longer period at their sub-inhibitory concentrations. Leakage of intracellular components followed by the loss of integrity of phospholipid bilayer and ultimately leading to cell death may be the reason for observed lesser bacterial density and deformed cells/debris. Licorice root, sage leaves, oregano, and thyme HWIs show bacteriostatic effects at lower concentrations, which suggest they may not have immediate cell wall or membrane destruction effect. The chemical structure of individual antimicrobial phytochemicals and their abundance in the efficacious HWIs are important for their mechanisms of action. Licorice root extract contains flavonoids, isoflavones, saponins, and coumarins, which may regulate the expression of genes responsible for bacterial virulence of streptococcus species have been reported [52,53]. Different species of thyme and oregano were reported to possess some antimicrobial activities [44]. Both of plant leaves or young shoots are used in various food preparations as flavor enhancers as well as in herbal remedies. Although the modes of action of the extracts are not clearly recognized, it may be due to the major bioactive compounds, including thymol, terpenes, eugenol, flavones, glycosides of phenolic monoterpenoids, and aliphatic alcohols, among others [44,54].

Herbal teas provide relief for pharyngeal inflammation in patients with streptococcal pharyngitis. Anti-inflammatory activities of herbal extracts and infusions have been shown by several authors previously [55,56]. Our recently published data on anti-inflammatory properties of these extracts have proven the ability of all four efficacious HWIs for suppression of pro-inflammatory cytokines released from inflamed human tonsil epithelial cells (HTonEpiCs) induced by antigens (lipoteichoic acid and peptidoglycan) of *S. pyogenes* [8]. Following the herbal treatment, production of IL-8, hBD-2, ENA-78, and CGP-2 was suppressed in HTonEpiCs. However, to use them as biomedicine such as herbal tea with antibacterial and anti-inflammatory compounds, the suppressive effect of pro-inflammatory cytokines production by HWIs is expected only to happen in inflamed cells without affecting to the healthy pharynx epithelium cells. Therefore, we have previously performed cell viability assay to assess the cytotoxicity of these extracts on HTonEpiCs and found that all HWIs showed no cytotoxicity to the HTonEpiCs in vitro [8]. Therefore, use of these HWIs seems to be safer but further validations using in vivo models are required. The dry weight-based extraction yield of licorice, barberry, oregano, and thyme were 19.5% 6.0%, 8.2%, and 18.6%, respectively [8] which could be further enhanced by the improved technology such as subcritical water extraction.

Moreover, the water-based extraction process used in this study is acceptable for functional beverage applications as no chemical was used in the preparation and allows incorporation of the extracts in natural health products without concern of potential toxic solvent residuals [57]. Additionally, the preparation of infusions is simple, fast, and economical, and their applicability in commercial product development is straightforward. Therefore, it is possible that the use of non-toxic concentrations of licorice, barberry, thyme, or oregano infusions to develop into functional beverages, biomedicine, and natural health products for the management of streptococcal pharyngitis.

## 5. Conclusions

We report the inhibitory effect on the growth and formation of biofilm of *S. pyogenes* by phytochemical-rich HWIs of selected Canadian traditional herbal plants. The water-based extraction process is economical, environment- and consumer-friendly as well as allows the incorporation of the extracts in functional foods without concern of solvent residuals. Therefore, we conclude that HWIs of licorice, barberry, thyme, or oregano at their non-toxic concentrations as safe and efficient symptomatic treatments for the management of discomfort conditions associated with strep throat of streptococcal pharyngitis patients. Potential mechanisms of antibacterial and antibiofilm properties of these efficacious infusions may be due to the direct destruction of cell wall/membrane, inhibition of biosynthesis of cell wall/membrane, and inhibition of protein synthesis involved growth, adherence, and biofilm formation. However, further investigations are required to determine the impact of HWIs on cell wall/membrane leakage and specific gene expression to reveal the mechanisms of cell disruption, adhesion, and biofilm formation by presumed phytochemicals.

## Figures and Tables

**Table 1:** Potential phytochemical profile of the selected herbal plant parts.

Plant Name		Family	Parts Used	Potential Major Phytochemicals ((M–H^+^)^−^, RT in min)	References
Common	Botanical				
Barberry	*Berberis vulgaris* L.	Berberidaceae	Roots	Gingerol (293.38, 8.06), Caffeic acid (179.16, 3.22), Naringin (579.54, 5.99), Naringenin (271.26, 7.32), and Rosmerinic acid (359.32, 2.01).	[12]
Clove	*Syzygium aromaticum* L.	Myrtaceae	Flower buds	Eugenol (163.20, 4.40), Eugenyl acetate (205.24, 8.43), and β-Ocimene (135.25, 4.25).	[13,14,15]
Echinacea	*Echinacea purpurea* L.	Asteraceae	Flowers	Caftaric acid (311.23, 2.44), Chlorogenic acid (353.31, 2.78), Caffeic acid (179.16, 3.27), Cynarin (515.46, 6.02), Echinacoside (785.73, 6.56), Cichoric acid (473.37, 4.78), and β-Sitosterol (413.70, 4.61).	[16,17,18,19]
			Stems	Quercetin (301.23, 6.12) and Eugenyl acetate (205.24, 8.43).	
			Leaves	Caftaric acid (311.23, 2.44), Cichoric acid (473.37, 4.78), and Caffeic acid (179.16, 3.27).	
Ginger	*Zingiber officinale* L.	Zingiberaceae	Rhizomes	Gingerol (273.38, 7.18), α-Humulene (203. 24, 2.50), Gingerol (293.38, 7.18), α-Thujone/β-Thujone/camphor (151.23, 4.35), α or β-Caryophyllene (203.35, 2.52), Caffetic acid (311.23, 10.07), and Liquirtin (419.39, 6.21).	[20,21]
Licorice	*Glycyrrhiza glabra* L.	Papilionaceae	Roots	Glycyrrhizin (821.94, 6.86), Glabridin (323.97, 7.54), Thymohydroquinone (165.22, 2.81), Naringenin (271.26, 7.32), Asparegene (131.12, 1.63), Liquirtin (417.39, 6.34), 5 -Methoxyhydnocarpin (493.45, 6.28), Cynarin (515.46, 6.02), Quercetin (301.23, 6.12), p-Cyemene (133.21, 0.82), Generdiol (153.23, 3.47), α-Humulene (203.35, 2.53), and Kaempferol (285.23, 6.42).	[22,23]
Oregano	*Origanum vulgare* L.	Lamiaceae	Flowering shoots	Rosmerinic acid (359.32, 6.20), Oleanolic acid (455.71, 7.23), ρ-Cymene (133.21, 1.04), and 5-Methoxyhydnocarpin (493.45, 6.25).	[24,25]
Olive	*Olea europeus* L.	Oleaceae	Leaves	Hydroxytyrosol, Rutin, Luteolin-7-glucoside, Oleuropein glucoside, Luteolin-4′-glucoside, Oleuropein, and Oleuropein aglycon.	[26,27,28]
Rose geranium	*Pelargonium graveolens* L.	Geraniaceae	Leaves	Geraniol (151.24, 6.62)	[29]
Sage	*Salvia officinalis* L.	Lamiaceae	Leaves	1,8-Cineole (153.24, 2.18 Borneol and/or Linalool and/or α-Terpineol and/or β-Pinene (153.24, 4.01), β-carotene (535.87, 3.46), γ-Terpinene and/or Mycrene and/or β-Pinene and/or α-Pinene (135.24, 4.24), Asparegene (131.12, 3.65), and α-Terpine (135.24, 4,42).	[25,30,31]
Slippery elm	*Ulmus rubra* Muhl.	Ulmaceae	Inner barks	Ursolic acid /Betulinic acid (455.71, 9.5) and β-carotene (535.87, 5.29)	[32]
Thyme	*Thymus vulgaris* L.	Lamiaceae	Flowering shoots	Thymol and Carvacrol (149.21, 6.65), Thymohydroquinone (165.22, 7.17), γ-Terpinene, Myrcene, and α-Pinene (135.24, 4.24), Gingerol (293.38, 7.68), and Kaempferol (285.23, 6.42)	[25,33]

(M–H^+^)^−^: deprotonated molecular mass; RT: retention time.

**Table 2:** The minimum inhibitory concentration (MIC) and minimum bactericidal concentration (MBC) of hot water infusions against three strains of *Streptococcus pyogenes*. ATCC: American Type Culture Collection.

Plant-Source, Plant Part	ATCC 19615			ATCC 49399			Clinical Isolate		
	MIC (mg/mL)	MBC (mg/mL)	MBC/MIC	MIC (mg/mL)	MBC (mg/mL)	MBC/MIC	MIC (mg/mL)	MBC (mg/mL)	MBC/MIC
Clove FB	12.50	25.00	2	12.50	25.00	2	12.50	25.0	2
Sage L	12.50	25.00		12.50	25.00		12.50	25.0	
Ginger-Canada Rh	50.00	>50.00	-	50.00	>50.00	-	NA	NA	-
Ginger-Chinese Rh	50.00	>50.00	-	50.00	>50.00	-	NA	NA	-
Oregano FB	3.13	6.25	2	3.13	6.25	2	3.13	6.25	2
Thyme FB	3.13	6.25	2	3.13	6.25	2	3.13	6.25	2
Licorice R	1.56	3.13	2	1.56	3.13	4	3.13	6.25	2
Barberry R	3.13	6.25	2	3.13	6.25	2	3.13	6.25	2
Echinacea L	50.00	>50.00	-	50.00	>50.00	-	NA	NA	-
Echinacea S	6.25	12.50	2	6.25	12.50	2	6.25	12.50	2
Echinacea F	50.00	>50.00	-	50.00	>50.00	-	50.00	>50.00	-
Geranium L	25.00	50.00	2	25.00	50.00	2	NA	NA	-
Slippery elm IB	>50.00	>50.00	-	>50.00	>50.00	-	NA	NA	-
Olive L	>50.00	>50.00	-	>50.00	>50.00	-	NA	NA	-
Penicillin G	0.0078	0.0156	2	0.0078	0.0156	2	0.0078	0.0156	2

FB: Flowering buds; F: Flowers; FS: Flowering shoots; Rh: Rhizome; R: Roots; IB: Inner bark; L: Leaves; NA: Not analyzed.

**Table 3:** Minimum biofilm inhibitory concentration (MBIC) against *Streptococcus pyogenes* strains.

Hot Water Infusions	MBIC (mg/mL)		
	ATCC 19615	ATCC 49399	Clinical
Licorice Roots	1.56 (1 × MIC)	6.25 (4 × MIC)	3.13 (2 × MIC)
Barberry Root	6.25 (2 × MIC)	6.25 (2 × MIC)	6.25 (2 × MIC)
Oregano Flowering shoots	6.25 (2 × MIC)	6.25 (2 × MIC)	6.25 (2 × MIC)
Thyme Flowering shoots	6.25 (2 × MIC)	6.25 (2 × MIC)	6.25 (2 ×MIC)
Penicillin G	0.0156 (2 × MIC)	0.0625 (8 × MIC)	0.0625 (8 × MIC)

MIC: Minimum inhibitory concentration.

## Supplementary Materials

The following are available online at https://www.mdpi.com/2227-9059/7/3/63/s1.
