# Supplementary material for: Herbal Tea for the Management of Pharyngitis: Inhibition of Streptococcus pyogenes Growth and Biofilm Formation by Herbal Infusions
Source: Biomedicines. 2019 Aug 24;7(3):63. doi: 10.3390/biomedicines7030063 (PMC6783935; doi:10.3390/biomedicines7030063)
Supplement: Supplementary file 1 [file biomedicines-07-00063-s001.pdf]

Supplementary Materials: Figure S1: S2 and S3.

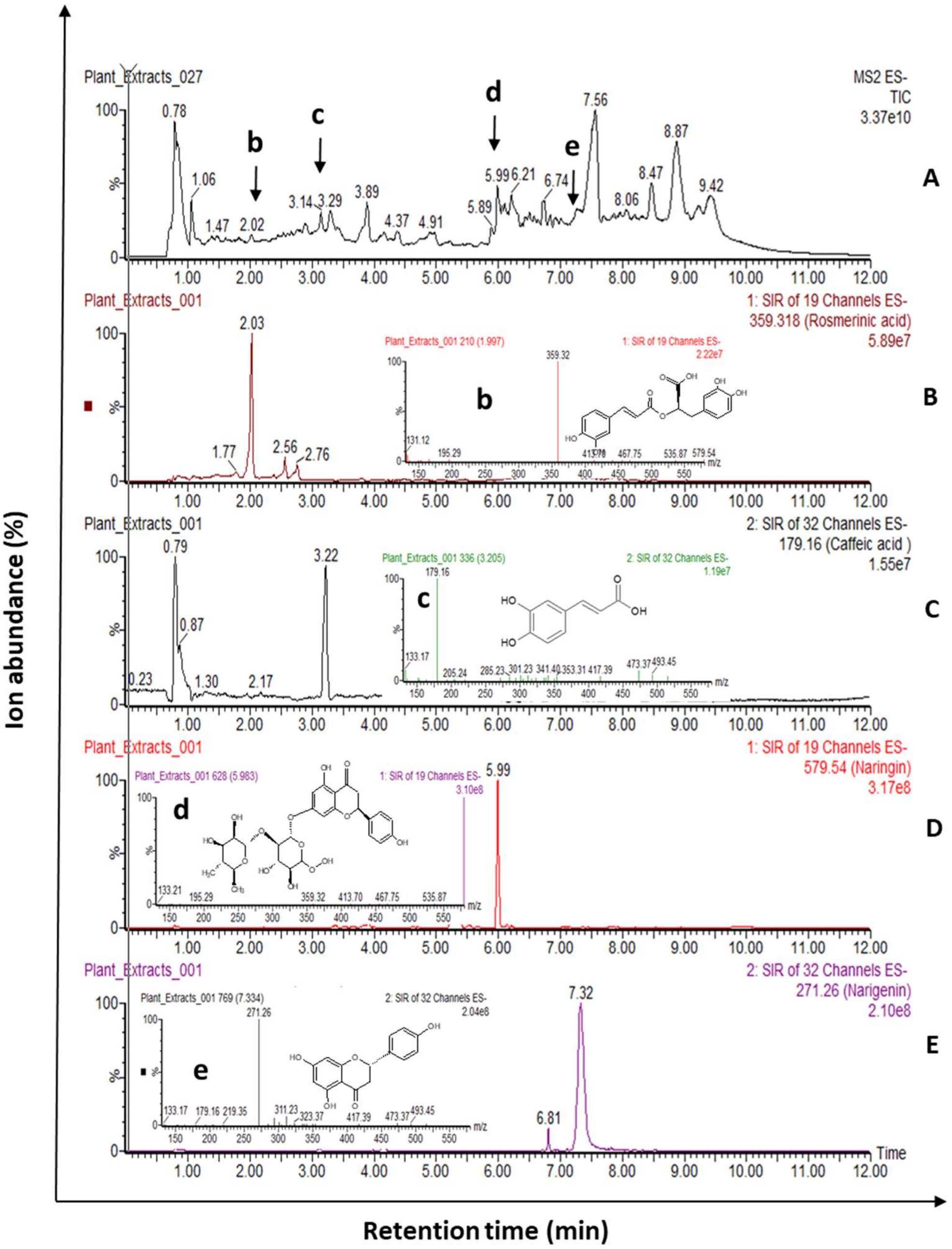

Figure S1. (A) The full scan mode total ion chromatogram of barberry root hot water infusion from a negative mode of PLC-ESI-MS/MS analysis. The SIM channels **(B,C, D, and E)** and mass spectra of the full-scan of selected four major phytochemicals (rosmarinic acid **[b],** caffeic acid **[c],** naringin **[d],** and naringenin **[e])** are shown for m/z of deprotonated ions of 359.32, 179.16, 579.54 and 271.26 respectively. The identified phytochemical name and chemical structure are presented. SIM: Selective ion monitoring scan; TIC: Total ion chromatograms, UPLC-ESI-MS/MS: Ultra-performance liquid chromatographic-electrospray ionization-tandem mass spectrometry.

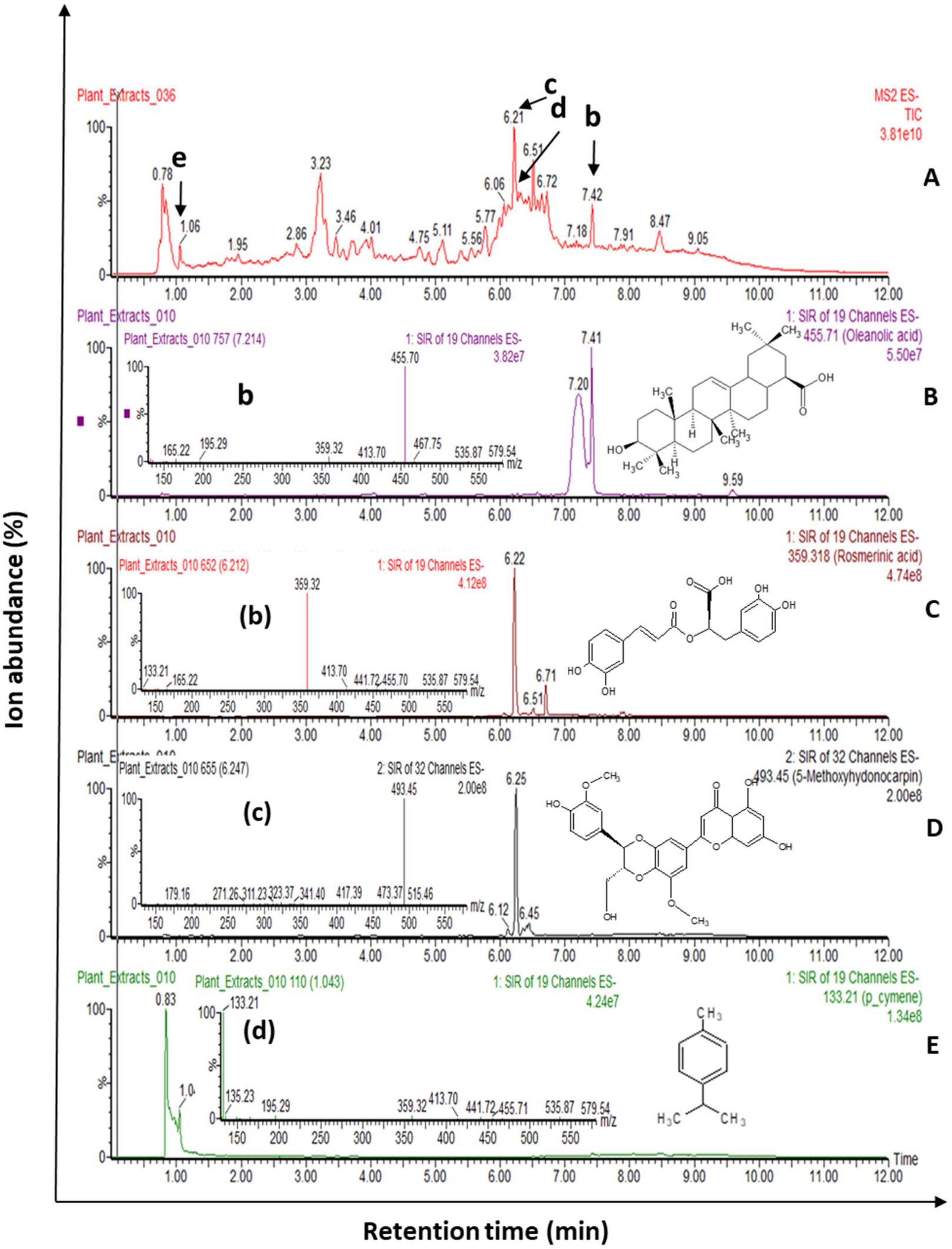

Figure S2. (A) The full scan mode total ion chromatogram of oregano flowering shoots hot water infusion from a negative mode of UPLC-ESI-MS/MS analysis. The SIM channels **(B, C, D, and E)** and mass spectra of the full-scan of selected four major phytochemicals (oleanolic acid **[b],** rosemarinic acid **[c],** 5-Methoxyhydnocarpin **[d],** and p-cymene **[e])** are shown for m/z of deprotonated ions of 455.70, 359.32, 493.45 and 133.21 respectively. The identified phytochemical name and chemical structure are presented. SIM: Selective ion monitoring scan; TIC: Total ion chromatograms, UPLC-ESI-MS/MS: Ultra-performance liquid chromatographic-electrospray ionization-tandem mass spectrometry.

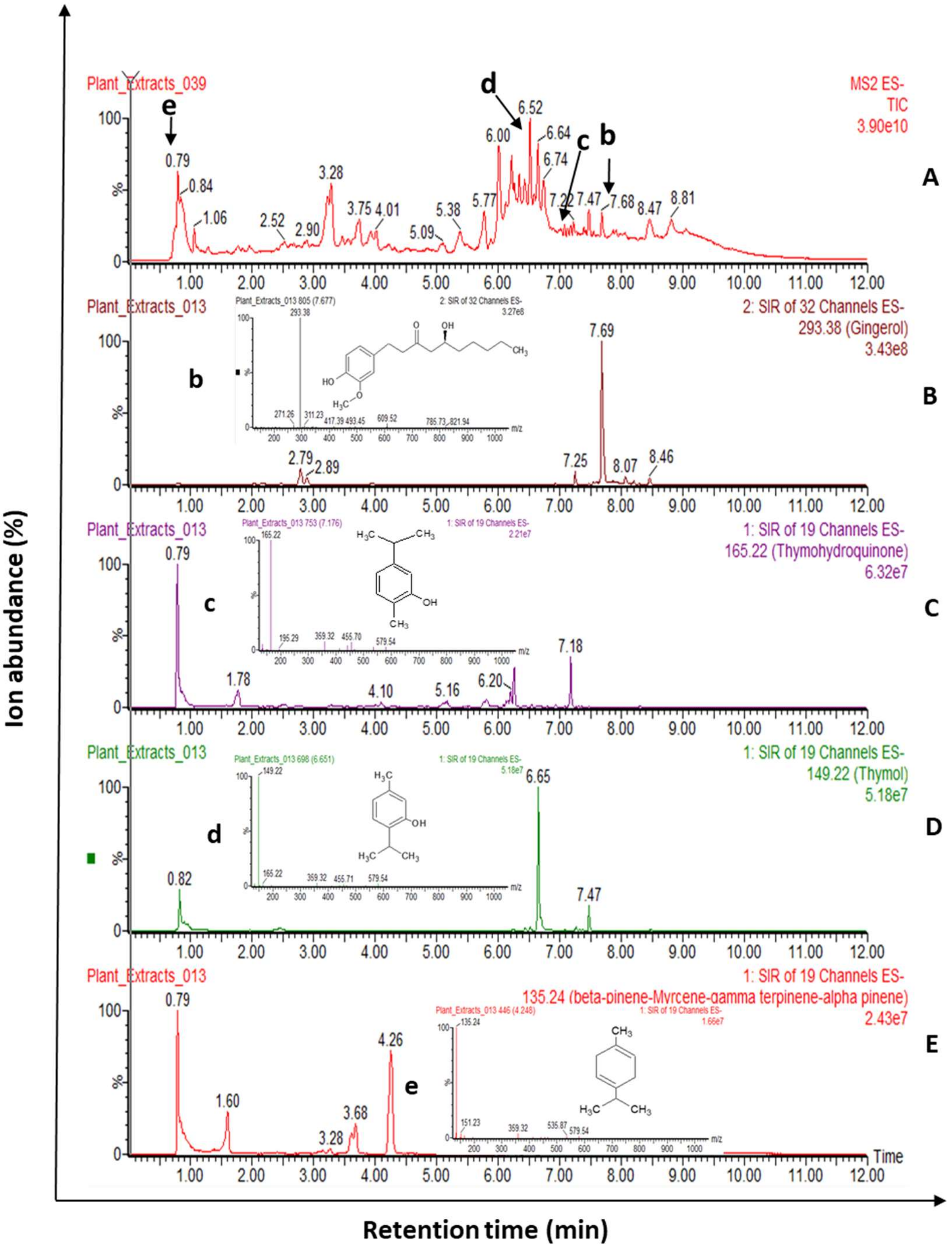

Figure S3. (A) The full scan mode total ion chromatogram of thyme flowering shoots hot water infusion from a negative mode of UPLC-ESI-MS/MS analysis. The SIM channels **(B, C, D, and E)** and mass spectra of the full-scan of selected four major phytochemicals (gingerol **[b]**, thymohydroquinone **[c]**, thymol **[d]**, and α-pinene **[e]**) are shown for *m*/*z* of deprotonated ions of 135.24, 149.22, 165.22 and 293.38 respectively. The identified phytochemical name and chemical structure are presented. SIM: Selective ion monitoring scan; TIC: Total ion chromatograms, UPLC-ESI-MS/MS: Ultra-performance liquid chromatographic-electrospray ionization-tandem mass spectrometry.
